# EEG‐guided anesthetic titration and perioperative cognitive disorders

**DOI:** 10.1002/alz.084904

**Published:** 2025-01-09

**Authors:** Lisbeth Anne Evered

**Affiliations:** ^1^ Weill Cornell Medicine, New York, NY, USA; St Vincent's Hospital, Melbourne, VIC Australia

## Abstract

**Background:**

Postoperative delirium (POD) is a serious complication of surgery associated with prolonged hospitalisation, long‐term cognitive decline, dementia and mortality. There is increasing evidence that electroencephalography (EEG) monitoring may reduce the incidence of POD, however, the best method for achieving this is unclear.

**Method:**

This presentation will present the results of a multicentre randomised clinical trial of 515 at‐risk patients undergoing major surgery from 8 centres in 3 countries who were assessed for POD for 5 days postoperatively. They also underwent cognitive screening at baseline, discharge, 30 days and one year postoperatively. Patients were assigned to light or deep anaesthesia. Additionally, this presentation will consider a meta‐analysis of available studies investigating EEG monitoring to prevent delirium and outline the importance of EEG spectrogram interpretation as part of EEG monitoring and consequent anesthetic titration when using this technique to reduce or prevent POD.

**Result:**

The incidence of POD was lower (19%) in the light anesthesia group compared with the deep anesthesia group (28%) (OR 0.58 (95% CI 0.38 to 0.88), P=0.010). Meta‐analysis results were fragile and depended on inclusion criteria of studies. Interpretation of EEG spectrograms is a developing field, but adds significant, critical information.

**Conclusion:**

EEG monitoring is a possible method for preventing POD and long‐term complications. EEG spectrogram interpretation will likely add critical information to enhance this technique.

